# Supplementary material for: Importance of the COVID-19 Vaccine Booster Dose in Protection and Immunity
Source: Vaccines (Basel). 2022 Oct 13;10(10):1708. doi: 10.3390/vaccines10101708 (PMC9610198; doi:10.3390/vaccines10101708)
Supplement: Supplementary file 1 [file vaccines-10-01708-s001.zip › vaccines-1927972-supplementary.pdf]

Table S1. The frequency of the type of covid-19 vaccine received.

	Whole sample N=176	BBIBP-CorV	ChAdOx1-S	rAd26-S/rAd5-S	CoVIran Barekat	Bharat Covaxin	BNT162b2
First dose	176	107 (60.8)	12 (6.8)	25 (14.2)	10 (5.7)	21 (11.9)	1 (0.6)
Second dose	173*	108 (62.4)	11 (6.4)	24 (13.9)	10 (5.8)	19 (11.0)	1 (0.6)
Third dose	64*	10 (16.4)	51 (83.6)	-	-	-	-

**3 cases had missing values; data are presented as number (percent)*
